# Diffusion and association processes in biological systems: theory, computation and experiment

**DOI:** 10.1186/2046-1682-4-2

**Published:** 2011-03-02

**Authors:** Paolo Mereghetti, Daria Kokh, J Andrew McCammon, Rebecca C Wade

**Affiliations:** 1 Heidelberg Institute for Theoretical Studies (HITS) gGmbH, Schloss-Wolfsbrunnenweg 35, 69118 Heidelberg, Germany; 2 Interdisciplinary Center for Scientific Computing (IWR), University of Heidelberg, Im Neuenheimer Feld 368, D-69120 Heidelberg, Germany; 3 Department of Chemistry & Biochemistry, Department of Pharmacology, and NSF Center for Theoretical Biological Physics, University of California San Diego, La Jolla, CA 92093-0365, USA

## Abstract

Macromolecular diffusion plays a fundamental role in biological processes. Here, we give an overview of recent methodological advances and some of the challenges for understanding how molecular diffusional properties influence biological function that were highlighted at a recent workshop, BDBDB2, the second Biological Diffusion and Brownian Dynamics Brainstorm.

## Introduction

In biological systems, macromolecules are constantly moving around by diffusion. How do the molecules find their binding partners? How do they fold to form a particular shape? How do they diffuse through the crowded environment of the cell interior? How does the presence of many diffusing macromolecules in a cell affect the function of the individual molecules? These are just some of the questions that are being pursued with the experimental and theoretical approaches that were discussed at the second Biological Diffusion and Brownian Dynamics Brainstorm (BDBDB2, http://mcm.h-its.org/BDBDB2) workshop. BDBDB2 was held on October 11-13, 2010 at the Heidelberg Institute for Theoretical Studies (HITS), with live evening videoconferencing sessions to the University of California, San Diego. The workshop brought together in Heidelberg about 40 theoreticians and experimentalists from around the world and a further 15 scientists in San Diego.

Brownian dynamics (BD) is a computational technique that allows the diffusive motion of molecules in solvent to be simulated, and a particular focus of the meeting was the discussion of current developments in this simulation methodology. Here we describe the main themes of the meeting, which provide a snapshot of the current state-of-the-art of studies of macromolecular diffusion, with a particular focus on BD simulation methods. We first discuss theories and methods for computer simulations for studying diffusional processes. In the next section, we describe theoretical and experimental studies on diffusion-influenced biochemical reactions. In the final section, experimental approaches for investigating diffusional processes *in vivo *are briefly described.

## Diffusional processes - Theories and Simulations

### Brownian and Langevin dynamics simulations

BD and Langevin dynamics (LD) simulation methods may be applied for the purpose of studying the motion and the interactions of biological macromolecules in solvent. The macromolecules are modelled as particles diffusing in a solvent that is modelled as a continuum that exerts frictional and random, stochastic forces on the particles. Common to these methods is the possibility of accessing phenomena whose time-scale is much greater than that usually achievable in atomistic molecular dynamics simulations. A range of methodological developments were presented at the BDBDB2 meeting and these have been implemented in a number of new software packages, as well as in existing packages. The approaches differ in the strategies used to minimize computational cost and, at the same time, retain sufficient detail and accuracy for studying the process of interest. The strategies used to extend the space and time-scale of the simulations can be divided into three categories: physical approximation, smart algorithms and parallelization. The different software packages take advantage of one or more of these strategies.

Many physical approximations have been described to reduce the complexity of the system simulated. When the internal degrees of freedom are not fundamental for describing the process studied, the macromolecules can be considered as rigid bodies. This approximation, which greatly reduces the complexity, allows the atomic details of the macromolecules to be retained. Atomically detailed rigid-body BD simulations have been implemented, for example, in Macrodox [1], UHBD [2] and SDA [3]. Paolo Mereghetti (HITS, Heidelberg, Germany) described extensions of the latter to the simulation of solutions of many protein molecules (with SDAMM) [4]. Adrian Elcock (University of Iowa, USA) described the ground-breaking application of this type of model to simulate a crowded cytoplasm-like environment made up of about fifty different types of macromolecules that occur in *Escherichia coli *[5,6].

One can further reduce the level of detail by keeping the rigid body representation and coarse-graining the atomistic details. For example, the representation of a molecule by a simple sphere with an excluded volume interaction or a sphere with a reactive patch interacting with a Coulomb potential, has been employed for analysing diffusional association processes [7].

In many cases, such as macromolecular folding processes or binding by induced fit or conformational selection, the rigid body approximation breaks down and a strategy that explicitly treats internal flexibility is required. A coarse grained representation is frequently used. Typically, groups of atoms are represented as beads interacting via a set of interactions that have been parameterized using more accurate methods or experimental information. Coarse grained models are implemented in the BD simulation codes: UHBD [2], BD_BOX (developed in Joanna Trylska's group at the University of Warsaw, Poland; unpublished), BrownDye [8], BrownMove [9] and Simulflex [10].

Smart algorithms are important for achieving computational efficiency. Gary Huber (University of California, San Diego, USA), for example, described several algorithms implemented in BrownDye [8], including an adaptive timestep procedure, charge lumping and a collision detection algorithm.

Parallelization and making use of state-of-the art hardware is equally important. In the BD_BOX software, Maciej Dlugosz (University of Warsaw, Poland) has made extensive use of GPU programming and parallel programming with the Message Passing Interface (MPI) and the shared memory openMP approaches. BD_BOX is intended to be an engine that allows the simulation of very large biomolecular systems treated as coarse grained polymers in implicit solvent.

In BD simulations, the solvent is treated implicitly, that is, the solvent granularity is neglected. In some cases, particular attention should be paid to the treatment of solvent-solute interactions. For example, Daria Kokh (Heidelberg Institute for Theoretical Studies, Germany) showed that, to properly describe the adsorption of proteins to metal surfaces with a continuum model using BD simulations, specific properties of the hydration shell on metal surfaces should be accounted for by including additional, semi-empirically parameterized terms in the protein-surface forces [11,12]. Often, hydrodynamic interactions (HI) are neglected. The question of the importance of HI, and how they can be treated in BD simulations, came up several times during the meeting and it will be discussed in the following section.

### The importance of the solvent: hydrodynamic interactions (HI)

Understanding the effects of HI on the diffusion and association of macromolecules in complex environments (e.g. within the cell) is non-trivial since the importance of HI strongly depends on the properties of the system itself (e.g. the dimension of the particles, covalent linking of particles, net charge, solute concentration, boundaries, etc.). Adrian Elcock found that HI are fundamental for reproducing experimental diffusional properties (translational and rotational diffusion) of flexible interconnected polymer chains by performing simulations of folding proteins with and without HI [13]. Moreover, Elcock demonstrated the importance of HI for the reaction rates computed using BD [6,14]. In particular, he showed that the absence of HI tends to contribute to the overestimation of the reaction rates [14].

For dilute solutions (< 0.1 volume fraction) of interacting unbound proteins, the effect of HI on diffusional properties is less crucial. Paolo Mereghetti showed how, in dilute regimes, the concentration dependent diffusion coefficient of lysozyme and BPTI solutions can be reproduced without explicitly including HI. These results agree with those obtained previously in similar simulations by McGuffee and Elcock [5]. Further, if one is only interested in equilibrium thermodynamic properties, HI do not play any role and can be neglected. Elcock showed how BD simulations without HI of a model of *E. coli *cytoplasm successfully describe the relative thermodynamic stabilities of proteins measured in *E. coli*.

Implementing HI in simulations is challenging as the canonical approach requires the factorization of a 3N × 3N diffusion tensor, which is an O(N^3^) problem (N is the number of particles). Efficient procedures to reduce the computational time were discussed by Jose Garcia de la Torre (University of Murcia, Spain) [15]; and Thiamer Geyer (Saarland University, Germany) described a new approximate method for computing the hydrodynamic coupling of the random displacements which scales as N^2 ^and is valid for HI that are not too strong [9,16]. This approach is advantageous for simulations since it reduces the cost of computing HI to the same order as the computation of the direct forces.

In a dense environment, the correct reproduction of dynamic properties can be expected to depend on accurate modelling of HI. Indeed, beside the far-field part of HI, usually modelled with the Rotne-Prager [17] Yamakawa [18] tensor, near-field many-body interactions, so called lubrication forces [19], become important. As shown by Gerhard Naegele (Institute of Solid State Research, Forschungszentrum Jülich, Germany), neglecting the near-field part leads to unphysical behaviour, such as negative sedimentation coefficients, or inaccurate estimates of diffusional properties [20,21]. To take care of both far-field as well as near-field HI, accelerated Stokesian dynamics, developed by Banchio and Brady [20], can be used. Recently, Ando and Skolnick performed Stokesian dynamics simulations of macromolecular motions in models of *E. coli *cytoplasm [21] and found the accurate treatment of HI important for reproducing measured protein diffusion coefficients.

### Continuum and hybrid methods

BD treats the primary solute species explicitly, and the solvent implicitly. That is, BD is based on a Langevin type formulation of time-dependent statistical mechanics [22]. As has been noted, this represents a coarse-graining of molecular dynamics type treatments, in which both the solute and solvent particles are commonly treated explicitly. An even greater degree of coarse-graining yields fully continuum level treatments of all diffusing solute and solvent species, corresponding to a Fokker-Planck type formulation of time-dependent statistical mechanics. The simplest example is the treatment of diffusing solutes in terms of the Smoluchowski diffusion equation, i.e. as a time-varying or steady-state concentration or distribution function that depends on spatial coordinates [23].

The continuum level treatments of diffusion have both advantages and disadvantages relative to BD treatments. Continuum level treatments offer computational efficiencies when very large numbers of simple (ideally, non-interacting, point-like) solutes are involved. Indeed, such descriptions are often amenable to analytical study. One familiar result is the Smoluchowski second-order rate constant for solute reaction with a perfectly absorbing, spherical target [24]. More complicated model systems can sometimes be dealt with by numerical solution of the relevant partial differential equations - the Smoluchowski equation or, for charged solutes, the Poisson-Nernst-Planck equation [25]. The numerical methods available include finite difference and finite element methods, among others [26]. Such methods have been applied, for example, to account for realistic anatomical structure in simulations of the diffusion of neurotransmitters in synapses [27] and of calcium ions in cardiac myocytes [28].

On the other hand, BD treatments offer advantages when the solute molecules are substantially non-spherical, are flexible, or have anisotropic interactions. The number of coordinates required to describe such systems in continuum terms grows rapidly as these factors are added in. Also, in the case of low concentrations of solute particles in critical regions, the Brownian treatments account for stochastic effects in the most natural way.

An appealing prospect for future work is the development of hybrid models, in which continuum type treatments can be used in some parts of space and BD treatments in other parts of space (e.g., in the region of an enzyme's active site, if stochastic effects may be important). An early effort in this direction has been described by the Helms group [29]. Another type of hybrid model that has proven very insightful utilizes a Fourier decomposition of continuous lipid bilayers plus Brownian timesteps to describe dynamical processes in biological membranes [30].

## Diffusion-influenced biochemical reactions

### Reaction rates

The speed of binding and reaction events is crucial to protein functionality in many biological processes. Bimolecular association kinetics can be represented by a two-step process with an intermediate state (AB)* referred to as a transient (or encounter) complex:

A+B↔(AB)*→C,

The first reaction step is driven by the relative diffusion of the molecules, A and B, and long-range electrostatic forces. It enables the binding partners to orient specifically and to go on to form a bound (native) complex, C [31]. When protein-protein association is limited by the first reaction step, the corresponding association rate constant is high (typically above ~10^5^-10^6 ^M^-1^s^-1^). Whereas slower binding is generally associated with conformational rearrangements of the binding partners during the second reaction step [32]. Among various biomolecular rate theories for modelling of diffusion-influenced reactions [33], Huan-Xiang Zhou (Florida State University, Tallahassee, USA) discussed the transient-complex theory. In this theory the rate of diffusion-limited protein-protein or protein-nucleic acid binding is computed by accounting for binding stereospecificity in BD simulations without intermolecular interactions and electrostatic interactions through Boltzmann averaging in the reaction region. This approach has been used to dissect the factors leading to high association rate constants of proteins [34] and to introduce mutations to make protein-protein binding quicker and tighter, e.g. for beta-lactamase and beta-lactamase inhibitor protein [32]. Gideon Schreiber (Weizmann Institute of Science, Israel) further showed from analysis of free energy landscapes for the latter two proteins that mutations stabilizing 'fruitful' rather than 'futile' encounter complexes increased the rate of association [35]. Barry Grant (University of California, San Diego, USA) applied BD simulations to predict the key residues for kinesin-tubulin association, the electrostatic enhancement of association rates, and the electrostatic biasing of the binding of kinesin to microtubules [36].

In an alternative approach, Martin Held (Free University, Berlin, Germany) showed how transition path theory [37] can be used to obtain the reactive pathway and rate constant of an association process, and described an application to the docking of small molecules to *E. coli *phosphate binding protein [38]. The method provides association dynamics and the binding mechanism, but is currently limited to a spherical geometry for the ligand and requires further extension to higher dimensional problems, such as protein-protein and protein-DNA binding.

### Effects of protein flexibility on the kinetics of diffusion-limited reactions

An important problem in the modelling of protein binding kinetics is the influence of protein conformational changes on the rate constant of the binding process. In particular, many enzymatic reactions cannot be understood from the rigid- protein viewpoint since conformational changes provide a mechanism for achieving enzyme specificity. This fact motivates development of kinetic models that take into account dynamic modulation of the active site accessibility. Andy McCammon (University of California, San Diego, USA) demonstrated how the kinetics of binding could be coupled to the kinetics of the conformational transitions of the protein by using a "gating" model [39,40]. Application to acetylcholinesterase has shown that intra-monomer gating of the substrate binding tunnel in acetylcholinesterase is a fast process (on the ps timescale) with the reaction rate limited by diffusion, whereas inter-monomer gating in the acetylcholinesterase tetramer is much slower (on the ns timescale) and modulates the rate of substrate binding [41]. The recent application of gating theory to the PBCV-1 mRNA capping enzyme [40], employing a combination of Brownian and molecular dynamics simulations, could be used to show that the mechanism of substrate binding was related to a "population shift" rather than an "induced-fit" model, and that the relative protein domain motions did not affect the rate of substrate binding.

An analytical approach to the binding rate constant problem for the "induced-fit" and "conformational selection" protein-ligand binding models was presented by Zhou [41]. He proposed that for any receptor-ligand complex, there is a continuum of binding mechanisms that are tunable by the timescale of the conformational transitions relative to the timescale of relative diffusion of the binding partners. As the rates of conformational changes in the receptor increase, the binding mechanism gradually shifts from conformational selection to induced fit.

### Diffusion-limited reactions in high-density and crowded surroundings

The widely used standard bimolecular kinetic relations are strictly valid only under dilute conditions and when the concentration of one component is considerably smaller than that of the other [32]. At high molecular concentrations, the diffusive characteristics are expected to be influenced by interactions between solute particles which may affect bimolecular rate constants. Schreiber and colleagues investigated the effect of changing the concentration of molecular crowding agents on protein-protein binding kinetics. Three characteristic kinetic regions were observed in experiments: low concentration, crowded, where the rate constant increases, and highly crowded, where the rate constant decreases back towards the low concentration level [42]. Interestingly, at crowding concentrations corresponding to those in the cell, the crowding agents had little effect on the protein-protein association rates and binding affinities. Gary Pielak (University of North Carolina at Chapel Hill, USA) has, however, found that protein crowders have a quite different effect from synthetic polymer crowders on protein rotational and translational diffusion rates. Using NMR relaxation data, Pielak and colleagues found that the difference was due to weak favourable, non-specific interactions between the protein crowders and the specific proteins monitored [43].

### Kinetic models of complex intracellular processes

A diverse range of methods is being developed for spatiotemporal modelling of multi-step cellular processes. Johannes Seibert (Free University, Berlin, Germany) described BD simulations to study the effects of membrane geometry on primary rod vision signal transduction. Protein diffusion and binding/dissociation processes in a disc vesicle of the primary rod for vision were studied by BD simulations of sphere models of rhodopsin and G-protein molecules.

Elfriede Friedmann (Heidelberg University, Germany) presented a numerical model with a mixed system of differential equations to investigate the effect of cell shape on the Janus kinase (JAK) signal transduction and activator of transcription (STAT) pathway in different cell types. A new numerical algorithm was introduced to reduce the long computational time caused by the fine mesh and small time step which were necessary due to the combination of fast diffusion with the slow activation and deactivation kinetics of STAT5 [44].

Johan Elf (Uppsala University, Sweden) discussed how different reactions may require different spatial or temporal discretization approaches. Specifically, processes in the reaction network cannot be considered on the same footing when macroscopic average rates in the coarse-grained states include many microscopic rebinding events between molecules. The latter reactions should be resolved in order to correctly calculate the probability of coarse-grained events. Elf described a new algorithm that allowed the transition to be made from the macroscopic to the microscopic limit in diffusion simulations and enabled a molecular level of process description [45]. The method was developed for the coarse-grained Reaction-Diffusion Master equation [46,47], a widely used framework for modelling intracellular reaction-diffusion processes involving many reactants. To obtain a correct solution in the microscopic limit, he proposed rescaling of the mesoscopic rate constant so that the equilibrium time would be the same in the master equation as in the microscopic model, thereby linking the microscopic and the coarse-grained framework.

## Diffusional processes *in vivo*

Considering macromolecular diffusion *in vivo*, so far, two studies have been carried out to simulate intracellular conditions at a molecular level of detail permitting direct comparison to experimental data. McGuffee and Elcock [6] and Ando and Skolnick [21] reproduced the *in vivo *translational diffusion coefficient of the green fluorescent protein, using simulations with two different detailed models containing a heterogeneous mixture of macromolecules from the *E. coli *cytoplasm.

Advances in techniques for single-molecule experiments are providing new insights into the diffusional properties of proteins in cells. Single-molecule kinetic experiments that allow the tracking of the free diffusion of a protein molecule in a living bacterial cell with millisecond time resolution and sub-diffraction spatial precision were presented by Johan Elf [48]. It was established in these experiments that, at least down to the 4 ms timescale, small protein molecules exhibit standard diffusion in the bacterial cytoplasm with translational diffusion coefficients of about a tenth their magnitude in water. Furthermore, by using a photo-activatable fluorescent probe to activate a few fluorescent molecules in a cell, it was possible to track the response mediator protein, RelA, and monitor its diffusion and (transient) binding to ribosomes. Similarly, Jim Weisshaar (University of Wisconsin-Madison, USA) described experiments monitoring GFP-labeled RNA polymerase diffusion in different parts of *E. coli *cells and the interaction with DNA transcription sites.

Fluorescence correlation spectroscopy (FCS) was used to study subdiffusive motion of proteins in different parts of the cell by Matthias Weiss (University of Bayreuth, Germany) and Joerg Langowski (German Cancer Research Center, Heidelberg, Germany). Anomalous subdiffusion due to crowding was found to be common, and also to affect the rates for protein-protein binding [49]. ^15^N in-cell NMR in *E. coli *was reported by Gary Pielak, suggesting that intrinsically disordered proteins in the cell have a different dynamical response to macromolecular crowding compared to structurally ordered proteins [50].

## Concluding Remarks

In this short overview, it has not been possible to cover all the interesting advances presented at BDBDB2, so we have aimed to highlight some of the main areas where rapid advances are being made, as well as some of the challenges that demand further development of both experimental and computational methods. From the computational and theoretical perspective, notable effort is being devoted to developing models to simulate *in vivo *conditions. Particular attention is being paid to hydrodynamic interactions and macromolecular flexibility. Experimentally, advances are, in particular, being achieved in single molecule kinetics and *in vivo *diffusion experiments. This snapshot of research on biological diffusion and BD simulations shows a vibrant field with growing synergy between experimental and computational studies from which many interesting results can be expected in the next few years.
